# Antimicrobial stewardship program (ASP): an effective implementing technique for the therapy efficiency of meropenem and vancomycin antibiotics in Iranian pediatric patients

**DOI:** 10.1186/s12941-019-0305-1

**Published:** 2019-01-29

**Authors:** Aliakbar Rahbarimanesh, Sayed Yousef Mojtahedi, Payman Sadeghi, Maryam Ghodsi, Sara Kianfar, Leila Khedmat, Saeedreza Jamali Moghadam Siyahkali, Mohammad Kaji Yazdi, Anahita Izadi

**Affiliations:** 1 Department of Pediatric Infection Disease, Bahrami Hospital, Tehran University of Medical Science, Shahid Kiaee Street (Ghasem Abad), Damavand Street, 1641744991 Tehran, Iran; 2 Department of Pediatric Nephrology, Bahrami Hospital, Tehran University of Medical Sciences, Tehran, Iran; 3 Bahrami Hospital, Tehran University of Medical Sciences, Tehran, Iran; 4 Department of Pediatric, Bahrami Hospital, Tehran University of Medical Sciences, Tehran, Iran; 5 Health Management Research Center, Baqiyatallah University of Medical Sciences, Tehran, Iran; 6 Ziaeian Hospital, Tehran University of Medical Sciences, Tehran, Iran; 7 Department of Pediatric Hematology and Oncology, Bahrami Hospital, Tehran University of Medical Sciences, Tehran, Iran

**Keywords:** Antibiotic therapy, ASP intervention, Antimicrobial management, Antimicrobial resistance, Meropenem, Vancomycin

## Abstract

**Background:**

Antimicrobial stewardship program (ASP) is a distinguished method to improve the prescription and efficacy of antibiotics.

**Aim:**

The efficacy of ASP and conventional methods was compared to measure the effectiveness of meropenem (MPM) and vancomycin (VMN) antibiotics in pediatric patients.

**Design:**

In an interventional quasi-experimental study, 135 children admitted in Children’s Hospital affiliated to University of Medical Sciences in time periods of 2014–2015 and 2015–2016 were assessed.

**Methods:**

The conventional and ASP methods in 2014–2015 and 2015–2016 were respectively utilized to provide the best antimicrobial therapy of MPM and VMN antibiotics in patient children. The data of mortality rate (MR), antibiotic prescription (AP), antibiotic dose (ADe), antibiotic duration (ADn), length of hospital stay (LOHS), and blood cultures (BCs) were compared across the years using the Chi square, independent *t* test, and Fisher’s exact test.

**Results:**

The levels of MR, AP, ADe, ADn, LOHS, and positive BCs using the ASP method in 2015–2016 were significantly lower those of in 2014–2015 using the conventional one (p < 0.05).

**Conclusions:**

The ASP method versus conventional one with a better efficacy can be employed as an antibiotic administration guide for MPM and VMN in the therapy of patients in community-based hospitals.

## Background

One of the most common therapeutic agents or active pharmaceutical ingredients prescribed in healthcare and medical services is antimicrobial drugs [1]. It was clearly reported that 30–67% of children in their length of hospital stay (LOHS) consumed at least one antimicrobial medicine [2]. The consecutive antibiotic therapy may thus result in adverse drug reactions leading to the increased mortality and morbidity rate, longer hospital stay and higher health costs [3]. Also, it may lead to the severe antibiotic resistance and nosocomial infections [4]. Antibiotic resistance is a growing problem worldwide [5], so that it has been estimated that about half of antibiotic prescriptions in medical centers are incorrect or unessential [6].

There are several well-known ways to decrease the antibiotic resistance such as hand washing, disinfection of hands and hospital tools, prompted laboratory tests, continuous medical education, vaccination, preparation of antimicrobial resistance files, and use of antimicrobial stewardship program (ASP) method [7, 8]. The last one is an optimal use of antibiotics including true antimicrobial drug with an appropriate antibiotic dose (ADe), and duration (ADn) [9–11]. This program was initially introduced in 1996 [12–14] aiming to the improvement in health care quality with better use of antibiotics, and subsequently the reduction of antibiotic resistance rate and health care costs [6, 15–17]. Hence, implication of this method and assessment of its practical outcomes are essential to establish a comprehensive guideline for patients’ healthcare with a minimized toxicity and bacterial resistance [18].

The risk of antibiotic resistance versus the beneficial utilization of antibiotics should be balanced [19]. The routinely antibiotic prescription is preferred among the patients and considered as a medical skill in physicians [20]. A lack in antibiotic prescription may result in legal issues for the physician’s [21]. Some strategies to implement the ASP protocol include educational programs, antibiotic order forms, computerized programs, automatic stop orders, antibiotic cycling, and step-down therapy [22, 23]. An ASP team includes seven parts such as infectious disease specialist, professional center of infection control, hospital manager, clinical pharmacist for infectious diseases, clinical microbiologist, epidemiologist, and information technology expert subject [24, 25].

Published literatures in pediatrics earlier demonstrated the optimistic effect of ASP implementation on the use of antimicrobial agents [26–31]. However, this technique has not become pervasive in Iran due to the lack of proper and organized implementation in public and private pediatric hospitals. Besides, the consumption of drugs especially antibiotics in Iran based on the available data is three times more than the mean global rate [32]. On the other hand, there is a need to utilize a successful and multidisciplinary ASP model because of the restricted availability of data related to pediatric infectious diseases and institutional resources. Nowadays, meropenem (MPM) and vancomycin (VMN), as well as ciprofloxacin are the most common used antibiotics in children hospitalized for a wide range of diseases and health disorders. As ASP as an innovative and strategic method in Iran has been lower applied to improve the prescription and efficacy of antibiotics, the efficacy of this method versus conventional one in the current study was compared for MPM and VMN antibiotics in Iranian pediatric patients to optimize infection-related patient outcomes.

## Materials and methods

### Study design and participants

**Fig. 1:**
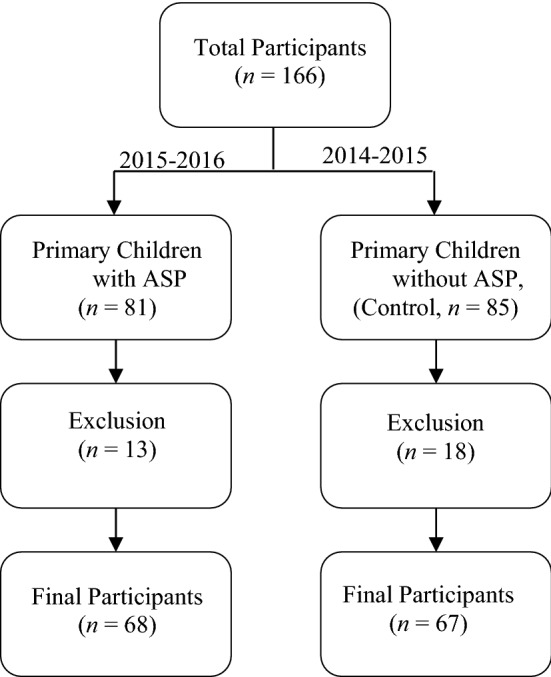
A schematic scheme of the whole and the adjusted comparisons of children groups studied in the current research

In this interventional quasi-experimental study, 135 children (mean age 12.2–20.6 months; mean weight, 7.8–8.7 kg) under treatment with MPM and VMN antibiotics in Children’s Hospital affiliated to University of Medical Sciences were eventually evaluated. The initial number of participants was 166. However, infants with a low birth weight or a LOHS lower than 1 week, birth at medical facilities other than NICUs, and having major congenital anomalies were excluded because most out-born infants after the first week of life were transferred and received variable nutritional feedings. In addition, several charts were missing key information, such as birth weight or discharge weight. Other exclusion criteria in this research were children referred to the surgery department and/or received antibiotics for prophylaxis. In general, 31 out of 166 people were excluded from the current study and (Fig. 1). Participation was completely voluntary and anonymous. Under parents’ consent, all obtained from questionnaire-based and clinical information were confidential and employed only for the blind study. We using a convenient and non-random sampling recognized all distinctive patients who received MPM and VMN antibiotics during two time periods from September 2014 to October 2016. The first period was considered as the 12 months before the ASP implementation from September 23, 2014 to September 22, 2015, while the second period was the first 12 months that the ASP was in place. This period was defined from October 23, 2015 to October 21, 2016. September 22, 2015–October 22, 2015 was also left out the research design as this time range was when the ASP was just getting begun.

### Antimicrobial stewardship program (ASP) setting

The core members of ASP team in this study was including specialist physicians and pharmacists in infectious diseases, skilled people in tracking outbreaks (epidemiologists), infection preventionists (IPs), clinical microbiologists, and two data scientists. From Saturday to Thursday, the ASP team officially inspects the primary instructions and applies daily and weekly usage arrangements of MPM and VMN. These antibiotics were purposefully prescribed according to the antimicrobial susceptibility patterns of clinical isolates or type of antibiotic-resistant infections, misapplication risk, and medical expenses. Besides, the ASP protocol incorporated pharmacist notification by personnel of clinical microbiology laboratory. After generating the real-time data collection, pharmacists present in the ASP team assessed each the documented order by evaluating the patient’s electronic medical record, subsequently issued some of key decisions/consultations after open communication with specialist physicians and sent a feedback to providers in terms of answers like stop, approve, and use of a sole antibiotic with a less/more dose.

### Data collection

An electronic and validated checklist including demographic (e.g., age, gender, and weight) and disease-related data were filled out for each patient and recorded in the pharmacy database. Clinical data included intensive care unit admission, disease type and intensity, antibiotic consumption history (such as receipt of past antibiotic therapy), ADe, ADn, LOHS, mortality, adverse effects, and results of bacterial cultures in MPM and VMN treatments, recommendations introduced by the ASP team about antibiotic prescription (AP) with a certain ADe, and patient’s agreement rate with consultations. The time difference (in day) between patient’s admission and discharge dates was considered as LOHS. The efficiency of used antibiotics against urinary tract infection (UTI), respiratory tract infection (RTI), and cerebrospinal fluid infection (CSFI) was respectively examined by the urine, tracheal, and cerebrospinal fluid (CSF) standard cultures. Gram staining from positive blood cultures was conducted to diagnose whether bacterial infections induced by Gram-positive cocci or by Gram-negative bacilli. The UTI was recognized from the clinical charts of all pediatric patients with substantial amounts of pathogens isolated in blood cultures. The UTI confirmation was also performed by checking their records to find the same pathogen in the urine and blood cultures, with pyuria by dipstick or sedimentary urine analysis and/or clinical symptoms (e.g., flank pain and fever) [33]. The diagnosis of lower/upper RTI was according to the following symptoms such as fever (T > 38.0 °C) or hypothermia (T < 35.5 °C), leukocytosis or leukopenia, and positive tracheal culture [34]. The CSFI was considered as a positive CSF culture with fever ≥ 38.0 °C [35]. In the ASP method, pathogenic results of early-positive blood cultures were quickly analyzed using a random-polymerase chain reaction (rPCR) [36, 37]. Thus, a couple of rPCR assays and BCs were determinant in the antibiotic continuation. The ADn was estimated from the beginning of appropriate antibiotic closest to collection date of BC. The stop date of scheduled antimicrobial treatment was also considered as the last day of appropriate antibiotics for patient’s children who were discharged alive.

### Statistical analysis

The data were statistically assessed using the analysis of variance (ANOVA) with SPSS software package, version 13.0 (SPSS Inc., Chicago, IL, USA). The independent *t*-student test and Chi square (χ^2^) or Fisher’s exact tests were performed to assess between-group differences for numerical and categorical variables, respectively. The significant level was set at p < 0.05.

## Results

**Fig. 2:**
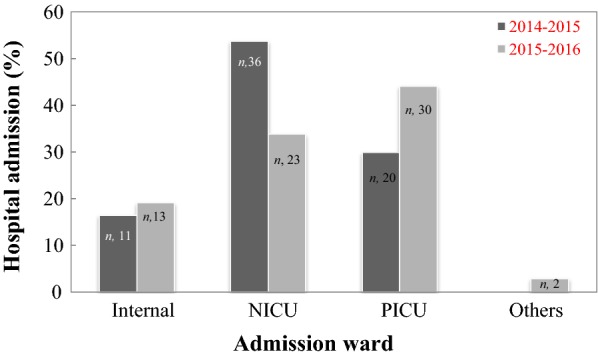
The admission percentage of pediatric patients (*n*, the patient number) in different wards of Bahrami Hospital during time periods of 2014–2015 and 2015–2016

Figure 2 depicts the number and percent of admission of patients in the various hospital wards such as internal medicine, neonatal (NICU), and pediatric (PICU) intensive care units and others. It can be seen in Fig. 2, the majority of patients were admitted in NICU and PICT wards. In details, the maximum admission was in the NICU (53.7%) in 2014–2015 and in the PICU (44.1%) in 2015–2016. The number of patient boys admitted in the time periods of 2014–2015 and 2015–2016 were 59.7 and 50.0%, respectively, whereas the corresponding number of patient girls was 40.3 and 50.0%, respectively (p > 0.05). Table 1 summarizes the frequency of 17 common diseases in in-patient children’s admitted in Bahrami Hospital in 2014–2016. This table reveals that the type of diseases was alike across the years (Table 1). Although pneumonia and sepsis in the investigated years were more prevalent diseases among the patient’s children, meningitis infection in 2015–2016 was highly detected (Table 1).

**Table 1:** The occurance rate of different diseases in in-patient children’s wards in 2014**–**2016

Disease type^a^	Considered time period			
	2014–2015 (non-ASP)		2015–2016 (with ASP)	
	Frequency (*n*)	Percent (%)	Frequency (*n*)	Percent (%)
Acute lymphocytic leukemia (ALL)	–	–	3	4.41
Acute gastroenteritis (AGE)	1	1.49	3	4.41
Left Congenital Diaphragmatic Hernia (CDH)	1	1.49	–	–
Bronchiolitis	2	2.98	–	–
Cerebral palsy (CP)	2	2.98	3	4.41
Leiomyosarcoma (LMS) tumor	2	2.98	–	–
Meningitis	2	2.98	7	10.29
Obstruction	2	2.98	2	2.94
Rheumatic fever (RF)	2	2.98	–	–
Tracheoesophageal (TE) fistula	2	2.98	–	–
Urosepsis	2	2.98	1	1.47
Posterior urethral valves (PUV)	3	4.47	–	–
Acute myeloid leukemia (AML)	4	5.97	3	4.41
Esophageal atresia (EA)	4	5.97	1	1.47
Necrotizing enterocolitis (NEC)	5	7.46	–	–
Pneumonia	7	10.44	9	13.23
Sepsis	14	20.89	16	23.53
Others	12	17.91	20	29.41

^a^The total number of children’s diseases in time periods of 2014–2015 and 2015–2016 was 67 and 68, respectively

The overall count of antibiotic prescriptions issued for the studied pediatric patients in time periods of 2014–2015 and 2015–2016 is given in Table 2. The antibiotic prescription was significantly reduced in 2015–2016 (p = 0.0001). Generally, a higher number (more than 3.4 times) of antibiotic prescriptions for VMN compared to MPM in 2014–2015 was issued. In addition, the percent of MPM and VMN prescriptions in 2015–2016 was significantly decreased from 10.44 to 1.47% and from 35.52 to 4.41%, respectively (Table 2). Interestingly, one patient who was treated in 2014–2015, did not experience any therapeutic adverse effect in 2015–2016 (p = 0.001). Also, the mortality rate was significantly reduced from 28.4% in 2014–2015 to 5.9% in 2015–2016 (p = 0.001). The results of different cell cultures for infectious diseases are also exhibited in Table 3. As shown in this table, a significant reduction in positive BCs from 2014–2015 to 2015–2016 was found (p = 0.001). Although a decrease in positive numbers of urine, tracheal, and CSF cultures was observed, this diminishing trend was not meaningful (Table 3). It is worth recalling that the implementation of ASP guideline could promote the management of UTI, RTI, and CSFI in pediatric patients. The ADe, ADn and LOHS amounts in 2015–2016 compared to 2014–2015 were significantly lowered using the ASP (Table 4).

**Table 2:** The total number of antibiotic prescriptions for studied pediatric patients

Time period	None	MPM	VMN	Both (MPM + VMN)
2014–2015 (without ASP)	–	7 (10.44%)^a^	24 (35.82%)^a^	36 (53.73%)^a^
2015–2016 (with ASP)	60 (88.23%)	1 (1.47%)^b^	3 (4.41%)^b^	4 (5.88%)^b^

Values in the same columns followed by different letters (a, b) are significantly different

Values inside and outside the parentheses respectively represent frequency (*n*, out of 67–68) and percent (%) of antibiotic prescriptions for the patient’s children

**Table 3:** The frequency of infectious diseases diagnosed in microbial culture laboratories

Culture type	Assessed period^b^		p value
	2014–2015 (non-ASP)	2015–2016 (with ASP)	
Blood culture^a^	16 (23.88%)^a^	3 (4.41%)^b^	0.001
Urine culture	5 (7.46%)	3 (4.41%)	> 0.05
Tracheal culture	2 (2.98%)	1 (1.47%)	> 0.05
Cerebrospinal fluid (CSF) culture	1 (1.49%)	–	> 0.05

^a^Values in the same row followed by different letters (a, b) are significantly

^b^The total number of tests in 2014–2015 and 2015–2016 was 67 and 68, respectively

**Table 4:** A summary of weight, age, and antibiotic dose and duration of therapy of patients stayed in the hospital in 2014–2016

Numerical variable	Studied period		p value
	2014–2015 (non-ASP)	2015–2016 (with ASP)	
Patient age (month)	12.2 ± 3.0	20.6 ± 6.5	> 0.05
Patient weight (kg)	7.8 ± 10.4	8.7 ± 6.7	> 0.05
LOHS (day)*	22.7 ± 1.9^a^	15.6 ± 2.8^b^	0.015
ADe (mg/kg)*	23.36 ± 3.8^a^	10.90 ± 3.6^b^	0.043
ADn (day)*	11.9 ± 9.5^a^	7.4 ± 4.6^b^	0.001

*LOHS* length of hospital stay, *ADe* antibiotic dose, *ADn* antibiotic duration

*Values in the same row followed by different letters (a, b) are significantly

## Discussion

The ASP method is a comprehensive approach to reduce the burden of the antibiotic resistance problem. This method as a practical strategy can notably improve the pattern and amount of antibiotic prescriptions in different hospital wards [38]. In this study, the outcomes of ASP and conventional method to control the antibiotic use of MPM and VMN in healthcare settings were compared in a community-based referral hospital in a developing country. Results showed that the ASP utilization not only can significantly reduce the ADe and ADn of MPM and VMN, but also can diminish the LOHS and number of positive cultures. Similar results were reported by Seah et al. [39], who studied the application effect of ASP method on the carbapenems use in a tertiary women’s and children’s hospital in Singapore. They found that the proper prescription of carbapenems using the ASP was increased from 55.9 to 70.3%. Also, the baseline ADe and ADn of carbapenems therapy were reduced up to 55.6 and 46.7%, respectively. Earlier, Di Pentima et al. [30] proved that the ASP protocol can be successfully applied to promote quality of care of hospitalized children by significantly decreasing targeted- and non-targeted-antimicrobial utilization up to 21%. Findings of Newland et al. [1] in the ASP efficiency assessment in pediatric hospitals in the United States were also in a good accordance with results of the present study. They noticed the higher antibiotic uphold rate with a monthly reduction rate of 7% during the ASP implementation compared to the control group. As well, the ADn and LOHS of antibiotic therapy were significantly reduced by applying the ASP. A significant decrease in ADn (33%) and LOHS (61–99%) for antibiotic treatments (e.g., ampicillin, cefotaxime, ceftazidime, eftriaxone, gentamicin, vancomycin, and MPM) was also reported by Lee et al. [40] in a tertiary children’s hospital after the guideline implementation of ASP. Song et al. [41] could successfully reduce the prescription of two antibiotics (~ 74%) against the infection of anaerobic bacteria by using the ASP method along with training course and inter-wards relationship enforcement. The considerable reduction in the antibiotic prescription was after the ASP utilization was comparable the antimicrobial efficiency of MPM and VMN in the present investigation. The prescription of a number of antibiotics including caspofungin, aztreonam, daptomaycin, ertapenem, voriconazole, linezolid, tigcycline, and MPM after the utilization of ASP method was assessed by Malani et al. [22]. Implementing the ASP led to a significant 50% decrease in the infection rate of *Clostridium difficile* with a reduced daily consumption dose (25.4%) and medical costs (15.2%). In the current study, the total dose of MPM and VMN was also reduced, but the calculation of costs was impossible.

Similar to results of our study, Morril et al. [42] reported the ASP was concomitant with a higher consultation count and a decreased time for consultation request leading to the improvement of patients’ health with a decreased rate of readmission and mortality. Thompson et al. [43] interestingly reported that use of ASP method would result in a better change from intravenous to oral antibiotic use. They achieved a significant success with an increased rate in oral antibiotics use. However, the pattern and route of antibiotic therapy were not considered in our study which may be a practical issue in further studies, especially in the pediatric population.

## Conclusions

In conclusion, because the ASP compared to the conventional method showed a superior effectiveness for MPM and VMN antibiotics, a culture-guided ASP is seriously proposed to community-based local hospitals owing to the assurance of implementation and performance of a proper antimicrobial therapy. A significant reduction in antibiotic consumption also can highly promote patients’ satisfaction and their expectations by decreasing medical costs and long-term disability for patients. Although the present research was limited by its quasi-experimental design, the obtained results in pediatric patients along with the evaluation of care quality can be verified by conducting further studies with larger sample size and multi-center sampling. Furthermore, the efficiency and satisfaction with the available ASP may potentially improve the therapy optimization by incorporating a prospective audit-and-feedback intervention.

## Abbreviations

ASP: antimicrobial stewardship program
MPM: meropenem
VMN: vancomycin
MR: mortality rate
APn: antibiotic prescription
ADe: antibiotic dose
AD: nantibiotic duration
LOHS: length of hospital stay
BCs: blood cultures
IPs: infection preventionists
UTI: urinary tract infection
RTI: respiratory tract infection
CSFI: cerebrospinal fluid infection
CSF: cerebrospinal fluid
rPCR: random-polymerase chain reaction
ANOVA: analysis of variance
